# Role of Natural Killer Cells in Uveal Melanoma

**DOI:** 10.3390/cancers12123694

**Published:** 2020-12-09

**Authors:** Asad Javed, Mohammed Milhem

**Affiliations:** 1 Department of Internal Medicine, Division of Hematology and Oncology, University of Iowa, Iowa City, IA 52242, USA; mohammed-milhem@uiowa.edu; 2 Holden Comprehensive Cancer Center, University of Iowa, Iowa City, IA 52242, USA

**Keywords:** uveal melanoma, NK cells, liver-resident NK cells

## Abstract

**Simple Summary:**

Metastatic Uveal Melanoma (MUM) is a lethal malignancy with no durable treatment available to date. A vast majority of patients with MUM present with liver metastasis. The liver harbors metastatic disease with an apparent lack of a cytotoxic T cell response. It is becoming evident that MUM is not an immunologically silent malignancy and the investigation of non-T cell anti-tumor immunity is warranted. In this review, we highlight the relevance of Natural Killer (NK) cells in the biology and treatment of MUM. Potent anti-NK cell immunosuppression employed by uveal melanoma alludes to its vulnerability to NK cell cytotoxicity. On the contrary, micro-metastasis in the liver survive for several years within close vicinity of a plethora of circulating and liver-resident NK cells. This review provides unique perspectives into the potential role of NK cells in control or progression of uveal melanoma.

**Abstract:**

Uveal melanoma has a high mortality rate following metastasis to the liver. Despite advances in systemic immune therapy, treatment of metastatic uveal melanoma (MUM) has failed to achieve long term durable responses. Barriers to success with immune therapy include the immune regulatory nature of uveal melanoma as well as the immune tolerant environment of the liver. To adequately harness the anti-tumor potential of the immune system, non-T cell-based approaches need to be explored. Natural Killer (NK) cells possess potent ability to target tumor cells via innate and adaptive responses. In this review, we discuss evidence that highlights the role of NK cell surveillance and targeting of uveal melanoma. We also discuss the repertoire of intra-hepatic NK cells. The human liver has a vast and diverse lymphoid population and NK cells comprise 50% of the hepatic lymphocytes. Hepatic NK cells share a common niche with uveal melanoma micro-metastasis within the liver sinusoids. It is, therefore, crucial to understand and investigate the role of intra-hepatic NK cells in the control or progression of MUM.

## 1. Introduction

Uveal melanoma is the most common intraocular malignancy and the second most common type of melanoma [1]. It occurs predominantly in the Caucasian population with an incidence of approximately 5–7/million/year in Europe and represents about 5% of total melanoma diagnosis in the United States [2,3]. Uveal melanoma originates from melanocytes within the uveal tract which comprises of the choroid, ciliary body and iris [4]. Primary uveal melanoma arises in the choroid in about 90% of the cases and of most patients initially present with visual symptoms [5,6].

## 2. Overview of Primary Uveal Melanoma Risk Stratification

In contrast to cutaneous melanoma, uveal melanoma has a distinct genetic and immunological profile despite their common melanocytic origin. 80–90% of uveal melanoma harbor mutations in the genes coding for G-protein-coupled receptor proteins *GNAQ* and *GNA11* [7,8]. A small subset of cases harbor mutations in *PLCB4* and *CYSLTR2* genes [9,10]. These mutations occur early during melanocytic malignant transformation. Subsequent chromosomal and genetic alterations broadly divide uveal melanoma into three metastatic-risk groups: (1) High-risk: Characterized by loss of one copy of chromosome 3 (Monosomy 3), gain of chromosome 8q and BRCA Associated Protein-1 (*BAP-1)* gene mutation leading to a loss of BAP-1 expression [11,12,13,14]; (2) Medium risk: Involving disomy 3, gain of chromosome 6p and *SF3B1* or *SRSF2* mutations [15,16,17]; (3) Low-risk: Involving disomy 3, gain of chromosome 6p and *EIF1AX* mutation [18,19]. Anatomic and histologic features of the primary tumor also predict metastasis such as large tumor basal diameter, tumor thickness, epithelioid histology, extra-scleral extension of tumor and ciliary body involvement [6]. 

Primary uveal melanoma is treated with the intention of limiting metastatic spread and preservation of vision. Treatment modalities commonly include radiation therapy (plaque brachytherapy, external beam radiation), laser therapy (trans-pupillary thermal therapy) and surgery. Surgery (enucleation) is performed in patients with vision loss, large tumor basal diameter or extra-scleral extension [20,21,22]. Approximately 50% of all primary uveal melanoma tumors have high-risk features and typically tend to develop clinical metastasis 2–3 years after initial diagnosis and treatment [23,24,25]. The most common site of metastasis is the liver [26]. Once metastatic disease develops, survival rate is dismal with a median survival of 6 months [27]. 

## 3. Limitations to Treatment of MUM

Treatment of MUM continues to be a challenge. Use of cytotoxic chemotherapy has demonstrated poor outcomes [28,29]. Surgical resection of liver metastasis has shown improved outcomes; however, surgery is less frequently utilized since MUM rarely presents as resectable oligometastatic disease [30]. Several forms of liver directed therapy have been investigated over the years including hepatic artery infusion, bland hepatic artery embolization, chemo-embolization, radio-embolization and embolization utilizing immune-adjuvant agents [31,32,33]. Limitations of liver directed therapy include restricted patient eligibility, invasiveness of the involved procedures, the potential for disruption of hepatic vasculature and the fact that the liver is not treated in its entirety and some form of systemic therapy is required to treat extra-hepatic disease. At best, liver directed therapy has shown modest improvement in survival in combination with systemic therapy [34]. Molecularly targeted systemic therapy in MUM has shown poor objective response rates and limited survival benefit [35,36,37]. 

Immune therapy has been extensively explored in MUM and continues to be investigated for its promise of a long-term durable response. Compared to cutaneous melanoma, MUM is poorly responsive to treatment with immune check-point inhibitors [38,39,40]. Recent advances in immune therapy in MUM include the use of adoptive transfer of tumor infiltrating lymphocytes (TILs) and novel soluble T cell receptor platforms. Clinical and objective responses seen with the adoptive transfer of TILs in MUM offers strong evidence that MUM is not ‘immune-refractory’. However, in clinical practice, its utility is limited by the processing time to treatment, low-yield of TIL extraction from MUM metastasis and increased toxicity from cytotoxic conditioning regimens [41]. Use of novel soluble T cell receptor platform (IMCgp100) is restricted for use in patients with specific Human Leukocyte Antigen (HLA) allotypes [42]. In summary, there continues to be an unmet need for the development of effective treatments for MUM. Specific to advancing immune therapy in uveal melanoma, it is crucial to explore non-T cell-based approaches. Table 1 provides an overview of current treatment options for MUM.

## 4. NK Cells: An Introduction

NK cells are a distinct subset of the immune system. In humans, NK cells comprise up to about 5–20% of the circulating lymphoid cells [43]. NK cells have been traditionally described as effector cells with a predominant innate immunological function. Without prior sensitization, NK cells can target transformed virus infected cells and tumor cells lacking Major Histocompatibility Complex (MHC) class I expression [44]. In addition to their innate function, NK cells also possess adaptive and memory like functions [45]. Unique subsets of NK cells have been identified within several tissues and organs. These tissue-resident NK cells exert local immunologic effector and regulatory functions relevant to their site of residence. A well described example is that of tissue resident NK cells in the pregnant uterus where they form the immunologic frontline at the maternal-fetal barrier [46]. 

Overall, NK cells offer a potent and broad repertoire of anti-tumor effector responses that can be therapeutically harnessed. Specific to uveal melanoma, NK cells offer an attractive treatment approach that is not directly T cell dependent. With an abundance of NK cells residing in the liver, we speculate that NK cells perform crucial immunologic functions within the complex microenvironment of uveal melanoma liver metastasis. In this review, we describe the role of NK cells in the control and progression of uveal melanoma.

## 5. Role of NK Cells in Primary Uveal Melanoma

### 5.1. NK Cell Suppression within Ocular Environment

The eye is an immune privileged organ which is well protected from generation of local inflammatory responses through several immunologic barriers. Protection from inflammation is critical to the functioning and survival of ocular corneal endothelial and retinal cells that are amitotic and lack the ability to regenerate [47]. In order to evade recognition and killing by cytotoxic T cells, normal ocular tissues express little or no classical MHC class I molecules [47,48]. This would make these tissues especially vulnerable to targeting by NK cells, but that is not the case. Within the eye, NK cell cytotoxicity is efficiently suppressed either by virtue of the immunosuppressive intra-ocular environment or through immunologic features of primary uveal melanoma tumor cells and its microenvironment. 

Aqueous humor of the eye contains immunosuppressive factors that directly inhibit NK cell function. Transforming Growth Factor-Beta (TGF-β) is found at a high concentration in aqueous humor [49,50]. TGF-β suppresses NK cell activation and function [51]. Paradoxically, TGF-β can also downregulate cell surface expression of MHC class I on intra-ocular tumor cells and make them more susceptible to NK cell lysis. However, at its relatively higher concentration within aqueous humor, suppression of NK cell function by TGF-β seemingly becomes the dominant factor in preventing tumor lysis [52]. Macrophage Migration Inhibitory Factor (MIF) is another immunosuppressive factor found within aqueous humor that protects corneal endothelial cells from NK cell mediated lysis [53,54]. Interestingly, MIF is also produced by uveal melanoma cells, enabling them to suppress NK cell function [55].

Suppression of NK cell anti-tumor response within the eye is well exemplified by the differential growth patterns of melanoma cells at intra and extra ocular sites, with restricted growth at extra-ocular sites and tumor progression within the eye [56,57]. In a mouse model study by Apte et al., a melanoma cell line which was susceptible to lysis by NK cells continued to progressively grow when implanted intracamerally into SCID mice (SCID mice lack T cell responses and have intact NK cell function) [57]. Similar results were seen in athymic mice. Melanoma growth was controlled when tumor cells were implanted into subcutaneous tissue. Tumor grew progressively at the subcutaneous sites when in-vivo NK cells were depleted. Exposure to ocular aqueous humor significantly inhibited NK cell lysis of melanoma cells in-vitro [57]. 

### 5.2. Infiltrating Immune Cells in Primary Uveal Melanoma

Contrary to other tumor types, the presence of infiltrating immune cells in primary uveal melanoma is a marker of poor prognosis [58,59]. Infiltrating immune cells in primary uveal melanoma include CD4^+^ T cells, CD8^+^ T cells [60], regulatory T (T_Reg_) cells [61,62], and Tumor associated Macrophages (TAMs) with the immunosuppressive M2 phenotype [59,63]. NK cell infiltration within primary uveal melanoma has not been as extensively investigated but appears to occur less frequently [59]. Cells with NK cell like activity and phenotype have been described in a minority (<2.5%) of TILs within primary uveal melanoma [60,64]. 

The inflammatory phenotype of primary uveal melanoma is associated with monosomy 3 and *BAP-1* loss that are known high-risk genetic occurrences in uveal melanoma predictive of metastasis [65,66]. It appears that infiltrating immune cells in uveal melanoma exert immunosuppression rather than being immunologically ineffective. For example, infiltrating CD8^+^ T cells in primary uveal melanoma assume regulatory rather than cytotoxic functions [67]. Recent studies have highlighted the association of loss of *BAP-1* with upregulation of immunosuppressive genes in primary and MUM [68,69]. In a study involving 32 different cancer types, Roufas et al. calculated and compared intra-tumoral immune cytolytic activity (CYT) [70]. CYT was calculated using the geometric mean of Granzyme-A and Perforin-1 toxins that are secreted by cytotoxic T cells and NK cells. Uveal melanoma exhibited the lowest CYT amongst the study tumor types [70]. TIGIT (T cell immunoreceptor with Ig and ITIM domain) is an immune-regulatory checkpoint receptor expressed on T-cells and NK cells [71]. TIGIT (along with CD96, a co-inhibitory receptor), competes with CD226 (an activating receptor) on T-cells and NK cells to favor immune suppression [72]. Blockade of TIGIT reverses NK cell exhaustion and promotes anti-tumor immunity [73]. Expression of TIGIT in uveal melanoma has been reported by Stalhammar et al. [74]. In their study, higher expression of TIGIT on intra-tumoral immune cells correlated with an increased risk of metastasis. The study authors observed that the number of intra-tumoral TIGIT positive cells outnumbered CD8^+^ T cells, indicating that other immune cells (including NK cells) might be suppressed within the tumor microenvironment via this checkpoint. 

### 5.3. NK Cell Suppression in Primary Uveal Melanoma Microenvironment 

Within the tumor microenvironment of primary uveal melanoma, numerous immunologic barriers offer protection to tumor cells from NK cytotoxicity. Tumor infiltrating immune cells and the cytokines they produce dampen NK cell effector function. Forkhead box protein P3 (FOXP3) positive T_Reg_ cells have been described in up to 24% of primary uveal melanoma tumors [61,62]. Specific to NK cells, production of TGF-β by T_Reg_ cells downregulates activating receptors on NK cells [75]. TGF-β2, an isoform of TGF-β, is upregulated in the eye under pathologic conditions [76]. TGF-β2 is also expressed in primary uveal melanoma and is indicative of tumor progression [77]. It is not clear whether the source of TGF-β in uveal melanoma is tumor cells or tumor infiltrating immune cells. M2 type Tumor associated macrophages (TAMs) are common in uveal melanoma [63], and they are known to produce TGF-β in other tumor types such as colorectal and lung cancer [78,79]. TAMs produce Interleukin-15 (IL-15) and uveal melanoma expresses IL-15 receptors [80]. Exposure of uveal melanoma cells to IL-15 causes proliferation of tumor cells and decreases the susceptibility of tumor cells to NK cell mediated cytolysis [80]. Interferon gamma (IFN-γ) is also highly expressed in aqueous humor of patients with uveal melanoma [81,82]. IFN-γ can play both tumor suppressive and tumor promoting roles in cancer [83]. It upregulates HLA expression in uveal melanoma causing indirect inhibition of NK cells. IFN-γ mediated upregulation of HLA is noted even in the presence of TGF-β (a down-regulator of HLA) [52]. IFN-γ also upregulates the expression of Indoleamine-2,3-dioxygenase (IDO) and Programmed Death Ligand-1 (PD-L1) which suppress NK cell activation [83,84,85].

The amino acid Tryptophan plays an important role in NK and T cell activation. Metabolism of Tryptophan is carried out via the Kynurenine pathway [86]. Kynurenine, which is a metabolite of Tryptophan plays an immune modulatory role by suppressing T cell and NK cell proliferation and promoting their apoptosis [87,88]. Tryptophan 2,3-dioxygenase (TDO) and IDO are rate limiting enzymes of the Kynurenine pathway [86]. Cancer cells can create an immunosuppressive microenvironment by upregulation of these enzymes, leading to Tryptophan depletion, Kynurenine accumulation and suppression of NK and T cell cytotoxicity and proliferation [89,90,91]. 

Uveal Melanoma does not constitutively express IDO in the primary or metastatic cells [88,92]. However, in the presence of IFN-γ, IDO expression is upregulated [92]. Once IDO is overexpressed, melanoma cells can then suppress local NK cell activation. Expression of prostaglandin-E2 and IDO by melanoma cells downregulates NK activating receptors (NKp30, NKp44 and NKG2D) [93,94]. 

Another mechanism utilized by NK cells to target tumor cells or infected cells is via death receptor-induced target cell apoptosis. NK cells and T cells express Tumor Necrosis Factor (TNF)-related apoptosis-inducing ligand (TRAIL) which binds to corresponding receptors on target cells to induce apoptosis [95,96]. Both primary and MUM cells express receptors for TRAIL [97]. In-vitro experiments have demonstrated susceptibility of uveal melanoma cell lines to TRAIL mediated apoptosis [97,98]. Interestingly, targeting of uveal melanoma via TRAIL has been shown to be significantly upregulated in the presence of interferon-Beta (IFN-β), highlighting the important role of biologic agents that could have therapeutic implications [97]. To counteract TRAIL mediated apoptosis, uveal melanoma cells upregulate expression of anti-apoptotic proteins such as Survivin [98,99].

**Figure 1:**
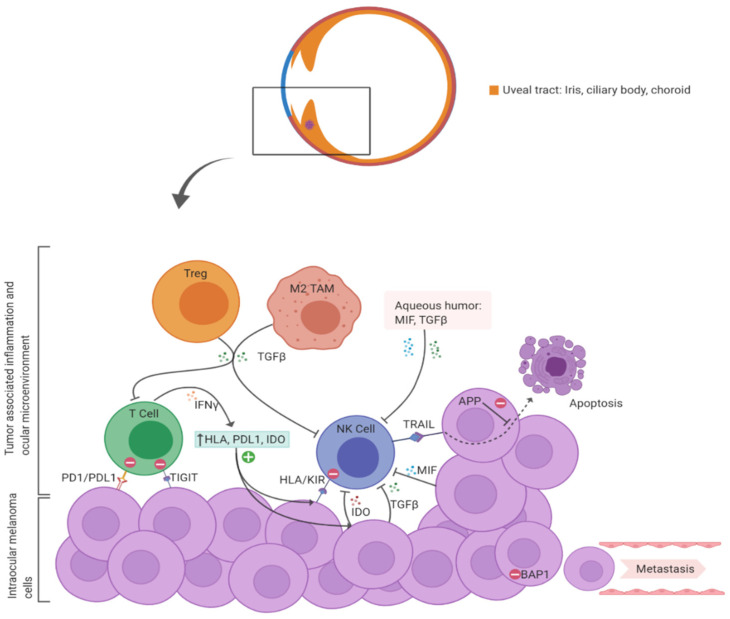
A diagrammatic representation of immune suppressive mechanisms within primary uveal melanoma and the ocular micro-environment that would inhibit NK cell function and cytotoxicity. APP: Anti-apoptotic protein; HLA: Human Leukocyte Antigen; IDO: Indoleamine-2,3-dioxygenase; IFN: Interferon; KIR: Killer Immunoglobulin-like Receptor (inhibitory receptor on NK cell that binds HLA on tumor cells); MIF: Macrophage Inhibitory Factor; TIGIT: T cell immunoreceptor with Ig and ITIM domain); TRAIL: Tumor Necrosis Factor (TNF)-related apoptosis inducing ligand on NK cells that binds receptors on tumor cells. (Figure created with BioRender.com).

To summarize, the presence of infiltrating immune cells in primary uveal melanoma correlates with intra-tumoral immunosuppression and poor survival. The apparent paucity of infiltrating NK cells in primary uveal melanoma is not surprising as intra-ocular and intra-tumoral microenvironments have potent immune-suppressive mechanisms in place to inhibit NK cell cytotoxicity. Outside of the immune privileged ocular environment, metastasizing melanoma cells would be especially vulnerable to circulating cytotoxic NK cells. Figure 1 outlines some of the mechanisms involved in suppression of NK cell function in primary uveal melanoma. 

## 6. Circulating NK Cell Control of Uveal Melanoma Metastasis

### 6.1. Role of Tumor HLA Expression

The eye lacks lymphatic drainage and uveal melanoma primarily metastasizes via the hematogenous route [100]. While in circulation, uveal melanoma tumor cells are susceptible to targeting by circulating immune cells. Metastasizing cells typically originate from high-risk tumors (with Monosomy 3, *BAP-1* loss), that are associated with tumor infiltrating immune cells [59,101]. A feature of primary uveal melanoma with an inflammatory phenotype (and high-risk for metastasis) is HLA class I expression on tumor cells [66]. Down regulation of HLA, particularly HLA class I, is a mechanism deployed by tumors to evade targeting by cytotoxic T cells whose T cell receptors can recognize and engage MHC class I expressed on tumor cells. In the case of uveal melanoma, metastasizing cells paradoxically upregulate cell surface HLA expression. It is believed that shedding of tumor cells with low HLA expression are detected and eliminated by circulating NK cells whereas the ones expressing HLA metastasize and survive. 

In-vitro studies have demonstrated the ability of cytotoxic NK cells to detect and kill uveal melanoma cells [52,57,102,103]. Moreover, animal model studies provide proof that NK cells play a crucial role in controlling metastasis, likely via systemic surveillance. In a study by Ma et al., mice were injected with melanoma cell lines with variable sensitivity to NK cell lysis [102]. Fewer metastasis occurred in the livers of mice receiving NK cell sensitive tumor cells. Abrogation of systemic NK cell function resulted in loss of anti-metastatic effect. Tumor cell lines that were insensitive to NK cell lysis demonstrated more liver metastasis with no change noted upon suppression of in vivo NK cell function. When an NK cell sensitive cell line was injected intracamerally, no liver metastasis was noted in mice following NK cell stimulation [102].

HLA expression by uveal melanoma cells correlates with an increased risk of metastasis and poor survival, likely due to tumor cells ability to evade recognition by circulating NK cells. Ma et al. showed that uveal melanoma cell lines that were sensitive to NK cell mediated lysis had reduced MHC class I expression [102]. In contrast, melanoma cells with normal MHC class I expression were insensitive to NK cell lysis both in vitro and in vivo [102]. In a subsequent study, uveal melanoma cell lines that were resistant to NK cell lysis were shown to have a high constitutive expression of MHC class I. When these cell lines were incubated in TGF-β (a down regulator of MHC class I on normal cells), significant reduction in MHC class I expression was noted as well as a corresponding increase in susceptibility to NK cell lysis [52]. A cell line which was sensitive to NK cell lysis with low MHC class I expression was incubated with IFN-γ (an up-regulator of MHC). This led to an increase in MHC expression by tumor cells and a corresponding decline in sensitivity to lysis by NK cells [52].

Blom et al. showed that expression of HLA-A and HLA-B in 30 primary uveal melanoma samples correlated with poor survival [104]. In fact, the prognostic impact of HLA-A expression was more significant than that of tumor diameter [104]. In a study of 65 primary uveal melanoma cases, higher expression of HLA class I and class II on uveal melanoma cells correlated with a significantly worse prognosis [105]. Interestingly, lower expression of HLA class I has been observed in uveal melanoma with spindle cell histology which is predictive of a favorable prognosis [106]. By upregulating HLA expression, it would be expected that circulating tumor cells would be more susceptible to targeting by cytotoxic T cells. However, uveal melanoma cells can maintain some level of HLA expression while at the same time undergoing HLA downregulation for certain HLA loci and alleles. That way it can potentially evade both T cell killing as well as NK cell targeting [107].

It is unclear as to how HLA expression is modulated in uveal melanoma. Evidence suggests that the external influence of IFN-γ, likely originating from infiltrating immune cells, serves as a potential trigger for HLA upregulation in uveal melanoma. Van Essen et al. studied the HLA regulatory system in uveal melanoma and determined it to be functional [108]. In 28 primary uveal melanoma samples, increased HLA protein expression correlated with increased transcription of peptide loading and regulatory genes. The studied regulatory genes were mainly related to interferon signaling. When tumors were implanted in SCID mice lacking an immune response, infiltrating lymphocytes were not seen, and HLA regulatory gene transcription was not enhanced, leading the study authors to conclude that increased HLA expression in primary uveal melanoma is most likely related to infiltrating immune cells [108]. 

### 6.2. NK Cell Ligand Expression on Uveal Melanoma Cells

Uveal melanoma cells have several cell surface receptors and ligands that facilitate interaction with NK cells. NK cells have a wide repertoire of activating and inhibitory receptors on their cell surface. These receptors interact with their respective ligands on both normal cells as well as target cells (tumor cells or virus infected cells). Generation of a net activating or inhibitory signal determines whether an NK cell will have a cytotoxic or tolerant effect [109]. One such diverse group of receptors found on human NK cells are the Killer Immunoglobulin-like Receptors (KIRs), including both activating KIRs and inhibitory KIRs (iKIRs). KIRs are highly polymorphic human NK receptors. For inhibitory KIRs (iKIRs), their ligands typically include HLA class I molecules: iKIR receptor KIR2DL1 binds HLA-C2, KIR2DL2–3 binds HLA-C1, KIR3DL1 binds HLA-Bw4 and KIR3DL2 binds HLA-A*03, A*011 [110]. Regarding activating receptors, some of the human NK receptors and their respective ligands are: NKG2D (Natural Killer, Group 2 member D) binding MIC A/B (Major histocompatibility complex class I chain-related protein A/B) and ULBP (UL16 binding protein) [111,112]; Natural Cytotoxicity Receptors (NCRs), NKp46 (CD335), NKp44 (CD336), NKp30 (CD337) [113]; and DNAM-1 (DNAX accessory molecule-1 or CD226) which binds CD112 and CD155 [114]. The entire repertoire of NK cell activating/inhibitory receptors and their corresponding ligands is extensive and continues to expand with ongoing research. Table 2 summarizes some of the commonly described NK cell activating and inhibitory receptors and their respective ligands. 

Uveal melanoma expresses ligands for NK cell receptors. Maat et al. showed the expression of several ligands for activating and inhibitory NK cell receptors in uveal melanoma cell lines, including HLA-A/B/C, ULBP1–3, MIC-A/B, CD155, CD112 [103] (Table 2). Interestingly, in primary uveal melanoma patients, the presence of homozygosity for HLA-C1 (ligand for iKIR KIR2DL2–3) and HLA-C2 (ligand for iKIR KIR2DL1) was associated with a worse melanoma related mortality as compared to patients who were heterozygous for HLA-C1/C2. A possible mechanism for NK cell protection from metastasis was proposed by the authors, suggesting that in the case of heterozygous HLA-C expression (C1/C2), loss of one HLA-C allele (C1 or C2) by the tumor will potentially lead to loss of cell surface expression of a ligand that will no longer be recognized by its respective NK cell iKIR. The resulting loss of inhibitory signaling will make the tumor cell more susceptible to lysis via net-activation of the NK cell [103]. The net cytotoxic effect of such a mismatch between NK cell inhibitory KIRs and their ligands has been clinically utilized in high risk Acute Myeloid Leukemia patients utilizing haploidentical KIR ligand-mismatched NK cells [115]. Apart from the classical HLA class I molecules, uveal melanoma is also known to express non-classical HLA class I, such as HLA-E [116]. HLA-E binds CD94/NKG2A (an inhibitory receptor complex) on NK cells, leading to NK cell suppression [117].

### 6.3. Potential Relevance of Chromosome 6 Aberrations

Specific to uveal melanoma, aberrations in chromosome 6 are a frequent occurrence in primary tumors. This is of particular relevance since the HLA complex is located on the short arm of chromosome 6. Several studies have shown improved survival in uveal melanoma patients with gain of 6p as compared to monosomy 3 [16,17,118,119]. Gain of 6p and monosomy 3 are typically mutually exclusive occurrences in uveal melanoma [120]. Evidence suggests that 6p gain does not lead to over expression of HLA [108], and that the protective effect from metastasis seen in patients with 6p gain could be instead due to the lack of presence of monosomy 3 (which is associated with intra-tumoral inflammatory infiltrate, increased HLA expression and poor survival). Loss of heterozygosity on chromosome 6p has also been reported in primary uveal melanoma without a correlate with HLA-A and HLA-B monomorphic expression [121]. As proposed by Maat et al., loss of expression of certain HLA alleles could lead to a mismatch between tumor cell surface HLA and iKIR on NK cells, potentially suppressing ‘net inhibitory’ signaling and favoring cytotoxicity [103].

## 7. Role of NK Cells in Uveal Melanoma Liver Metastasis

### 7.1. Uveal Melanoma Dormancy of Hepatic Micro-Metastasis

Despite effective local control of tumor recurrence with surgery or brachytherapy, a significant proportion of primary uveal melanoma patients develop metastatic disease [122]. Metastasis occurs via hematogenous route and the liver is the most common, and often the first site of metastasis [26,100]. This is an intriguing metastatic presentation, since circulating tumor cells encounter pulmonary capillary beds prior to metastasizing to the liver. Uveal melanoma cells are known to express certain receptors (c-Met, IGF-IR, CXCR4) for which corresponding ligands are expressed in the liver (HGF, IGF, CXCL12) [123]. This could explain the homing of circulating tumor cells to the liver. Alternatively, uveal melanoma cells that originate in the immune privileged ocular environment are preferentially able to survive and grow in the immune-tolerant liver.

Predictive mathematical models of uveal melanoma tumor progression indicate that metastasis occurs several years prior to clinical diagnosis of the primary tumor [124,125]. This means that liver metastasis could occur in the early stages of growth of the primary tumor. This hypothesis was supported by Borthwick et al. who studied autopsy specimens from patients with primary uveal melanoma [126]. A subset of the studied patients had died due to non-melanoma related causes. Interestingly, single cells or small micro-metastasis were noted in the livers of patients who were asymptomatic from melanoma at the time of their death [126]. 

Following metastasis, single tumor cells or micro-metastatic clusters appear to remain in a state of dormancy [127,128]. Autopsy studies in uveal melanoma patients indicate that hepatic micro-metastatic tumor deposits are present mainly within the sinusoidal spaces and they outnumber larger tumor masses [129,130]. Uveal melanoma micro-metastasis lacks presence of vascularization and demonstrate low cellular proliferation. Moreover, there is an absence of an associated inflammatory response with hepatic micro-metastasis, indicating that the ‘growth arrest’ of micro-metastatic tumor cells is unlikely to be immunologically mediated [126,130]. 

Work by Grossniklaus et al. presents two distinct patterns of uveal melanoma metastatic growth in the liver: infiltrative and nodular [129,130]. Metastasis with an infiltrative growth pattern seems to be the more prevalent presentation. Infiltrative growth pattern metastasis originates within the sinusoids and then expands to form larger metastatic masses that are more vascular and proliferative. Interestingly, CD56^+^ NK cells are present within the sinusoids along with the infiltrative pattern metastasis, but do not seem to occur as a part of an inflammatory response. On the other hand, nodular pattern metastasis tends to be localized adjacent to portal venules. Nodular foci of metastasis express Vascular Endothelial Growth Factor (VEGF) and are associated with CD3^+^ T cells [129,130].

### 7.2. Evidence for Role of NK Cells in Controlling Uveal Melanoma Liver Metastasis

Multiple in-vivo murine model studies have demonstrated the efficacy of NK cells in controlling the extent of uveal melanoma liver metastasis as well as the pattern of metastatic spread. In a study by Dithmar et al., C57BL/6 mice received intracameral injections of B16-LS9 melanoma cells [131]. Two groups of mice received different durations of neoadjuvant intramuscular recombinant human interferon alfa-2b injections; a control group received none (recombinant interferon was used with the rationale that it augments anti -tumor NK cell activity [132]). All mice underwent enucleation of the affected eye and were prospectively evaluated for occurrence of metastasis. None of the mice receiving recombinant interferon developed liver metastasis. Mice receiving a longer duration of interferon did not develop any lung metastasis [131]. In a subsequent study, Yang et al. investigated intracameral injections of three different melanoma cell lines with varying degrees of HLA class I expression using a similar experimental design [133]. Recombinant human interferon alfa-2b was once again used in the neoadjuvant setting. The authors demonstrated that treatment with interferon lowered murine liver micro-metastasis with a corresponding increase in hepatic NK cell mediated tumor apoptosis [133].

Alizadeh et al. reported their findings on mice with intraocular melanoma receiving intraocular and intravenous injections of adenovirus vector carrying the IFN-β gene [56]. In-vivo NK cell activation was demonstrated, and it correlated with enhanced clearance of liver metastasis as compared to the control group. Elimination of in-vivo NK cells abrogated the anti-metastatic effect, proving that enhanced NK cell function was contributory towards the therapeutic effect [56]. Yang et al. investigated the role of Entolimod in murine model of metastatic ocular melanoma [134]. Entolimod (CBLB502) is an agonist of Toll-like receptor (TLR). Activation of TLR leads to signaling via Nuclear Factor Kappa B (NFKB) pathway which in turn upregulates production of inflammatory cytokines and influx of various types of immune cells [135]. Mice with intraocular tumors received Entolimod vs placebo. Entolimod administration led to decreased frequency of liver metastasis. Moreover, it caused an enhanced homing of circulating NK cells to the liver as well as their maturation and activation [134].

As mentioned previously, Grossniklaus et al. described the infiltrative and nodular growth patterns of uveal melanoma metastasis in the human liver [129,130]. Like human autopsy cases, a higher ratio of infiltrative to nodular metastatic pattern was reported in murine metastatic ocular melanoma models [136]. Abrogation of NK cell activity led to an increase in number of liver metastasis as well as an increase in the proportion of larger metastatic deposits. Moreover, in the absence of NK cells, there was a shift in the pattern of liver metastasis with a relative abundance of nodular pattern metastasis indicating that NK cells control growth patterns of liver metastasis.

It should be noted that in most of the above mouse model studies, cutaneous melanoma cell lines were used. In particular, the B16-LS9 cell line has low HLA class I expression and is more susceptible to NK cell lysis [56,133,134,136]. NK cell stimulating agents such as recombinant interferon typically has a low half-life so larger systemic doses in human studies would carry a higher risk of toxicity [137]. While these mouse model studies certainly do not replicate the human ocular melanoma metastatic microenvironment, results of these studies are hypothesis generating and provide a proof of concept for the role of NK cells in controlling intrahepatic melanoma metastasis.

## 8. Tumor Specific Suppression of NK Cells in Uveal Melanoma Metastasis

Similar to primary uveal melanoma, infiltrating immune cells are present in uveal melanoma liver metastasis [138]. Infiltrating immune cells in MUM appear to have abundance of tumor associated macrophages and variable numbers of TILs [139]. Specific to infiltrating lymphocytes, studies have mainly described presence of T cell subsets and infiltrating NK cells in MUM has not been formally investigated [41,139,140,141]. Amongst CD3^+^ T cells in MUM, CD8^+^ T cells seem to be present at the interface of tumor and normal liver whereas CD4^+^ T cells are more concentrated in the perivascular areas [139]. Interestingly, the degree of infiltrating CD8^+^ T cells is comparable between uveal melanoma and cutaneous melanoma metastasis [140]. The presence of CD3^+^, CD4^+^ and CD8^+^ T cells within metastasis is also similar when specifically comparing liver metastasis of uveal and cutaneous melanoma [141]. Despite the numerical comparability, only a small proportion of CD8^+^ T cells from uveal melanoma metastasis can be successfully cultured ex-vivo. Overall, TIL cultures from uveal melanoma liver metastasis show limited anti-tumor reactivity as compared to cutaneous melanoma liver metastasis [141]. Indeed, compared to cutaneous melanoma metastasis, MUM has significantly lower expression rates of PD-1 on infiltrating lymphocytes [140,142]. Within cutaneous melanoma metastasis, liver metastasis have the lowest expression rates of PD-1 on infiltrating lymphocytes and PD-L1 on tumor cells as compared to other metastatic sites, suggesting site specific immune regulatory role of the liver. Taken together, anti-tumor immunologic responses in MUM are dampened by virtue of the immunosuppressive aspects of the tumor as well as due to hepatic immune tolerance. Cytotoxic NK cells infiltrating the tumor or in proximity would be susceptible to this immune suppressive environment.

MUM cells replicate many of the anti-NK cell responses that are present in primary uveal melanoma. Like high-risk primary uveal melanoma, expression of MHC class I in MUM cells has been reported [141,143]. According to Rothermel et al., MHC class I expression was noted in 75% of examined MUM cases [141]. The proportion of uveal melanoma metastasis expressing MHC class I was comparable to what was noted in cutaneous melanoma liver metastasis [141]. Expression of MHC class I would lower the susceptibility of metastatic cells to NK cell targeting. It is unclear whether MHC expression is constitutively upregulated by uveal melanoma metastasis or if it is a result of infiltrating immune cells in larger metastatic deposits, like what is observed in primary tumors [108]. MHC expression on uveal melanoma micro-metastasis during its dormancy has not been described, such information will be useful to predict the susceptibility of uveal melanoma micro-metastasis to NK cell lysis. 

NK cells and T cells express a potent activating receptor, NKG2D. NKG2D receptor binds MHC class I related ligands on target cells that are upregulated under conditions of cellular stress. In humans, ligands for NKG2D include the non-classical MHC molecules MIC-A, MIC-B and the UL16-binding proteins, ULBP1–4 [144,145]. MIC-A, MIC-B and ULBP1–3 have been reported in uveal melanoma cell lines [103]. Vetter et al. reported MIC-A and MIC-B expression in 50% of primary uveal melanoma tumors [146]. MIC-A/B expression correlated with NKG2D expression on TILs. None of the studied MUM tumors expressed MIC-A or MIC-B and none of the TILs in metastasis expressed NKG2D. The study suggests that expression of ligands for NKG2D is suppressed in uveal melanoma metastasis, or tumor cells that lack expression for NKG2D ligands are selected to survive and proliferate [146]. Downregulation of NKG2D on tumor infiltrating NK cells also occurs in hepatocellular carcinoma and liver metastasis of colorectal cancer, along with their suppression of cytotoxic and proliferative potential [147]. 

As previously described, MIF is an NK cell inhibitory cytokine that is produced by normal ocular tissue as by primary uveal melanoma cells [53,54,55]. Repp et al. showed that MIF was also produced by MUM cell lines. Moreover, metastatic cell lines produced about twice the amount of functional MIF as compared to primary uveal melanoma cell lines [55].

Although the immunosuppressive enzyme IDO is not constitutively expressed by liver metastasis, tryptophan 2,3-dioxygenase (TDO) (which along with IDO, is a rate limiting enzyme of the Kynurinine pathway) is widely expressed in the liver [86]. Terai et al. reported expression of TDO in uveal melanoma liver metastasis, with higher expression noted within tumor metastasis as compared to liver parenchyma [88]. Expression of TDO was also demonstrated in MUM cell lines. Thus, the liver facilitates production of tryptophan metabolites that suppress NK cell and T cell functions and MUM cells seem to be more potent producers of such immunosuppressive metabolites. 

## 9. Hepatic Immune Tolerance and Its Role in Suppressing NK Cell Function

The human liver can be described as a lymphoid organ with a plethora of lymphocytic populations including T cells, B cells, NK cells, gamma-delta T cells, NKT cells and Innate lymphoid cells [148,149]. Despite the abundance of immune cells, the liver favors tolerance over reactivity. The liver is a highly vascular organ with about 80% of its circulating blood originating from the portal system which is rich in antigens from food and gut microbes. Portal blood eventually circulates in the liver sinusoids where the sluggish blood flow allows for ample interaction of these antigens with lymphocytes and macrophages (Kupffer cells) within the sinusoids and the space of disse. It is crucial for the hepatic immune system to process and eliminate antigens locally without generating a broader cytokine response that would lead to inflammation and tissue injury. Clinically, hepatic tolerance is exemplified by superior allograft survival in liver transplantation as compared to other solid organ transplants [150,151]. This immune tolerance of the liver is exploited by viruses (Hepatitis B, Hepatitis C), hepatic parasitic infections (malaria) and tumors (Hepatocellular carcinoma and liver metastasis). 

**Figure 2:**
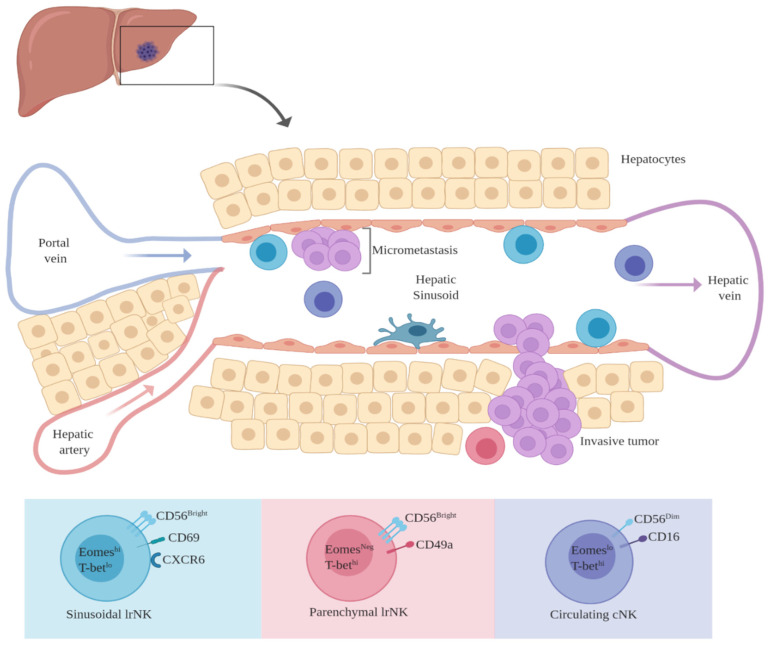
Phenotypic diversity of hepatic NK cells and their potential for interaction with intra-sinusoidal micro-metastatic disease. Liver resident (lr) NK cells are non-circulating cells found within hepatic sinusoids and parenchyma. Conventional NK cells (cNK) are freely circulating within the hepatic vasculature. (Figure created with BioRender.com).

It is highly likely that within the hepatic sinusoids, uveal melanoma micro-metastatic foci survive within an immune tolerant niche that is maintained both by tumor cells and liver cells. Hepatic sinusoids represent an immunological buffer between portal blood and the liver parenchyma. Several non-parenchymal hepatic cells reside within and around the sinusoidal space including Kupffer cells, liver sinusoidal endothelial cells, hepatic stellate cells and hepatic dendritic cells (Figure 2). These non-parenchymal cells also perform multiple immunologic functions, overall favoring tolerance rather than reactivity. Under physiologic conditions, kupffer cells exposed to bacterial lipopolysaccharide induce tolerance by production of IL-10 [152,153]. Liver sinusoidal endothelial cells (LSECs) are known to be potent inducers of T_Reg_ cells through TGF-β [154,155,156]. T_Reg_ cells, in-turn, exert immune suppression by producing IL-10 and TGF-β, which can dampen the cytotoxicity of infiltrating and circulating NK cells. FOXP3, a marker of T_Reg_ cells has been demonstrated in uveal melanoma liver metastasis [157].

## 10. Liver Resident NK Cells: Their Potential Immunomodulatory Role in MUM

### Hepatic NK Cells: Conventional vs. Liver-Resident NK Cells

Although it is beyond the scope of this review, it is Important to highlight the role of hepatic NK cells in immunity and homeostasis. NK cells comprise less than 20% of the total circulating lymphocytes. In peripheral blood, a vast majority (>90%) of NK cells are characterized as CD3^-^ CD16^+^ CD56^Dim^ with high cytotoxicity potential. A small subset of circulating NK cells is CD3^-^ CD16^-^ CD56^Bright^ and these cells are believed to be precursors to the CD56^Dim^ circulating NK cells. CD56^Bright^ NK cells in peripheral blood have poor cytotoxic potential but are efficient at cytokine production such as IFN-γ [158].

Like peripheral blood, subgroups of NK cells are also present within solid organs. Overall, there seem to be two distinct, non-overlapping subsets of NK cells within solid organs: The freely circulating or ‘conventional’ NK cells (cNK) and tissue resident NK cells. Tissue resident NK cells have been described in several organs including the liver, uterus, salivary gland, adipose tissues, and kidneys [159]. An interesting example is that of uterine-resident NK cells, which represent the predominant leukocyte population of the pregnant uterus. Uterine resident NK cells play a crucial role during pregnancy by maintaining immune tolerance at the maternal-fetal interface, avoiding fetal rejection and promoting vascular remodeling by producing angiogenesis promoting factors [159,160]. The human liver has a vast repertoire of lymphoid cells. Hepatic NK cells account for almost 50% of the total hepatic lymphocytic population [149]. Subsets of hepatic NK cells are differentiated based on phenotypic markers of tissue residency and by the expression patterns of certain T-box transcription factors, most notably T-bet and Eomesodermin (Eomes) [161]. 

The human liver has at least three subsets of NK cells (Figure 2): (1) conventional NK cells (cNK cells): These are the freely circulating NK cells within the hepatic vasculature that are phenotypically similar to CD3^−^CD16^+^CD56^Dim^ NK cells in peripheral blood. Transcriptionally, cNK cells are Eomes^Lo^T-bet^Hi^. They lack markers of tissue residency and likely perform functions like their circulating counterparts [162,163,164]; (2) CD56^Bright^ CD49a^−^, non-circulating, liver resident (lr) NK cells. These NK cells typically reside within the hepatic sinusoids. They express markers of tissue residency (CD69, CXCR6) and lack markers of egress from the liver. Transcriptionally they are categorized as Eomes^Hi^ T-bet^Lo^. They are proficient at degranulation, are less efficient at producing pro-inflammatory cytokines and are known to express high amounts of TRAIL. They are believed to be long term residents within the liver, surviving for several years and are possibly replenished from circulating precursors [162,163,165,166]; (3) CD56^Bright^CD49a^+^ lr NK cells. Transcriptionally, these NK cells are Eomes^Neg^T-bet^Hi^ and are non-overlapping with the CD56^Bright^Eomes^Hi^T-bet^Lo^ sinusoidal NK cells. These NK cells are found within the liver parenchyma. They represent a minority of the total hepatic NK cell population although they seem to proliferate during hepatic inflammation. They express high levels of activating NK receptors, as well as MHC class I specific receptors. They show lesser degree of degranulation but are potent producers of IFN-γ, TNF and GM-CSF (Granulocyte-macrophage colony-stimulating factor) [162,167,168].

Overall, hepatic NK cells have diverging and dynamic immunologic functions. Their role in liver cancer and liver disease continues to be explored and defined. Available evidence suggests that lr-NK cells could either have an immune-regulatory role in cancer progression or are present as hypofunctional, exhausted cells in advanced hepatic cancer. NK cell infiltration within advanced liver cancers has been described. Easom et al. demonstrated tumor infiltrating NK cells with a liver resident phenotype (CXCR6^+^CD69^+^) in Hepatocellular carcinoma (HCC) and colorectal cancer liver metastasis [147]. The authors identified these phenotypically liver-resident NK cells to be the predominant tumor infiltrating NK cell population. Tumor infiltrating NK cells had poor cytotoxic function with down regulation of NK cell activating receptor (NKG2D) as compared to non-tumor hepatic NK cells and circulating NK cells. Interestingly, this effect was reversible by in-vitro exposure to NK cells to IL-15 [147]. Pugh et al. showed that NK cells represented about one third of tumor infiltrating lymphocytes in colorectal cancer liver metastasis [169]. Infiltrating NK cells had a tolerogenic phenotype (NKG2A^Hi^KIR^Lo^) and their intra-tumoral presence correlated with inferior clinical outcomes. The authors did not classify NK cells as being liver resident or not [169]. Wu et al. showed that the presence of functional intra-tumoral NK cells in HCC correlated with an earlier stage of disease and improved survival [170]. More advanced stage HCC was associated with lower numbers of infiltrating NK cells that were exhaustive and hypo functional. The authors did describe studied NK cells as CD56^Bright/Dim^ but further characterization of hepatic CD56^Bright^ NK cells was not done [170]. In HCC, the presence of tumor infiltrating CD49a^+^ NK cells with an exhaustive, immune regulatory phenotype correlated with poor survival [171]. There is also accumulating evidence that lr-NK cells play a role in viral hepatitis and hepatic inflammation. NK cells are known to eliminate anti-viral cytotoxic T cells during chronic hepatitis infection [172]. Specific to lr-NK cells, mouse model studies have highlighted the ability of NK cells to suppress T cell anti-viral function and restricting T cell proliferation in autoimmune cholangitis [173,174]. It is, therefore, important to further explore the role of lr-NK cells in hepatic oncology and disease. It is becoming clear that hepatic NK cells represent a diverse population of NK cells with distinct immunologic functions. 

Investigating the role of lr-NK cells could be crucial towards a better understanding of uveal melanoma liver metastasis. Uveal melanoma micro-metastasis tends to survive within the hepatic sinusoids [129]. This is a remarkably intriguing presentation since these ‘dormant’ metastatic cells share a common space with sinusoidal lr-NK cells, potentially for years. Additionally, within the sinusoids, tumor cells are continuously exposed to circulating conventional NK cells and cytotoxic T cells, yet they remain undetected and survive. Evidence suggests that liver derived TFG-β helps maintain the CD56^Bright^Eomes^Hi^T-bet^Lo^ phenotype of lr-NK cells [175]. The vast majority of lr-NK cells have the CD56^Bright^Eomes^Hi^T-bet^Lo^ phenotype and these CD56^Bright^ NK cells reside predominantly within hepatic sinusoids [162,176]. CD56^Bright^ lr-NK cells would therefore be present within close proximity to uveal melanoma micro-metastasis. 

It also appears that sinusoidal circulating cNK cells seem to have a lower potency to kill despite the higher likelihood of them interacting with uveal melanoma micro metastasis within the sinusoidal space. In general, NK cells that express inhibitory receptors (iKIRs) specific for MHC class I are ‘educated’ or primed to kill target cells lacking MHC class I. NK cells that are constitutively lacking expression of inhibitory receptors are likely to undergo anergy or exhaustion and would be hyporesponsive to cellular targets [177,178,179]. Burt et al. demonstrated that hepatic CD16^+^CD56^Dim^ cNK cells have a significantly lower ability to kill MCH class I deficient targets as compared to their CD16^+^CD56^Dim^ counterparts in peripheral blood [180]. Both liver cNK and peripheral blood CD16^+^CD56^Dim^ NK cells had similar expression of activating NK cell receptors and similar quantities of intracellular perforin and granzyme B. However, hepatic cNK cells had a lower expression of MHC class I specific inhibitory receptors (KIRs and NKG2A), meaning that hepatic cNK cells seem to lack optimal priming to kill target cells with low MHC class I expression. The study authors were able to demonstrate that under appropriate pro-inflammatory in-vitro conditions (increased Interleukin-2), the cytotoxicity of hepatic NK cells could be enhanced [180]. The factor of low expression iKIR expression by lr-NK cells is especially highlighted in the case of CD56^Bright^ lr-NK cells that seem to completely lack KIR expression [162]. Interestingly, and potentially more relevant to hepatic NK cell targeting of cancer cells, the parenchymal CD56^Bright^CD49a^+^Eomes^Neg^T-bet^Hi^ subset of lr-Nk cells express MHC class I specific receptors (KIR, NKG2C) and are able to efficiently produce high levels of pro-inflammatory cytokines (IFN-γ, TNF and GM-CSF) [167]. Indeed, it is this CD56^Bright^CD49a^+^Eomes^Neg^T-bet^Hi^ subset of lr-NK cells that seems to proliferate and expand during inflammatory liver disease [168]. Potential anti-tumor cytotoxicity of this subset of lr-NK cell should be further investigated. Figure 2 summarizes the phenotypic characteristics of hepatic NK cells and their potential interaction with and proximity to intra-sinusoidal micro-metastasis. 

## 11. Future Direction

There continues to be an unmet need for exploring novel therapies in MUM. Regarding an NK cell-based therapeutic approach, ample pre-clinical data warrants further investigation in MUM [56,57,102,131,133,134,136]. At present, there are no active clinical trials investigating the therapeutic potential of NK cells specifically in uveal melanoma. NK cell immunotherapy is a rapidly developing field and its application in the treatment of MUM is relevant. Immuno-therapeutic modalities involving systemic administration of cellular NK product or enhancement of anti-tumor NK cell cytotoxicity have been investigated in several types of malignancies, including cutaneous melanoma. Table 3 summarizes current NK cell-focused immunotherapy clinical trials enrolling cutaneous melanoma patients. Similar immunotherapy approaches can be explored in MUM. Furthermore, it remains to be determined whether the vast array of intra-hepatic resident NK cells play a role in the progression or control of uveal melanoma metastasis. Considering their involvement in liver disease and inflammation, it is less likely that hepatic NK cells are immunologically silent in MUM. Investigating lr-NK cells in the context of MUM will be insightful towards a better understanding of the disease and development of novel therapeutics.

## 12. Conclusions

MUM continues to be a difficult disease to treat. Available evidence underscores the important role of NK cells in surveillance and targeting of metastasizing uveal melanoma cells in circulation. This role of NK cells potentially creates opportunities for treating intra-hepatic metastatic disease through amplification of NK cell function. The high propensity of uveal melanoma to metastasize to the liver, an organ with a dense and diverse NK cell population adds to the relevance of NK cells in control or progression of MUM. Further investigation of the role of NK cells in uveal melanoma liver metastasis is warranted.

## Figures and Tables

**Table 1:** Current therapies used in the management of MUM.

Liver-Directed Therapy	Systemic Therapy
Embolization: chemotherapy, immunotherapy, radio-embolization, bland embolization	Consideration for clinical trial
Ablative procedures	Immune checkpoint inhibitors
External beam radiation therapy	Cytotoxic chemotherapy
Surgical metastasectomy (in select cases)	Targeted therapy

**Table 2:** Relevant NK cell activating, and inhibitory receptors and their corresponding ligands expressed in uveal melanoma.

Common Human NK Inhibitory Receptors (iKIRs) and Their Corresponding Ligands	Common Human NK Activating Receptors and Their Corresponding Ligands	NK Ligands Expressed in Uveal Melanoma and Their Corresponding NK Receptors
KIR2DL1 (HLA-C2) KIR2DL2–3 (HLA-C1) KIR3DL1 (HLA-HLA-Bw4) KIR3DL2 (HLA-A*03, A*011) CD94/NKG2A/B (HLA-E) [110]	NKG2D (MIC A/B, ULBP) DNAM-1 (CD112, CD155) NCRs: NKp46, NKp44, NKp30 (Heparan Sulfate Glycosaminoglycans and others) [111,112,113,114]	HLA-A/B/C (iKIRs) HLA-E (NKG2A) ULBP1–3 (NKG2D) MIC-A/B (NKG2D) CD155, CD112 (DNAM-1) [103,116]

DNAM-1 (DNAX Accessory Molecule-1); HLA (Human Leukocyte Antigen); KIR (Killer Immunoglobulin-like Receptor; MIC-A/B (Major histocompatibility complex [MHC] class I chain-related protein A/B); NCR (Natural Cytotoxicity Receptor); NKG2A/D (Natural-killer group 2, member A/D); ULBP1–3 (UL16 binding protein 1–3).

**Table 3:** Current clinical trials using NK cell based immune therapy in cutaneous melanoma.

Clinical Trials.Gov ID	Study Drug(s)	Mechanism of Study Agent Utilizing NK Cell Anti-Tumor Effect
NCT03841110 Phase I Advanced malignancy (Including melanoma)	FT500 Pembrolizumab Atezolizumab Nivolumab IL-2	FT500 is an allogeneic, off the shelf, NK cell product derived from induced pluripotent stem cell (FT500 administered either as monotherapy, in combination with check point inhibitor, or in combination with check-point inhibitor and IL-2). Study drugs include fludarabine and cyclophosphamide as lympho-conditioning agents
NCT04592653 Phase II Advanced malignancy, including cutaneous melanoma	ALKS 4230 Pembrolizumab	ALKS 4230 is an engineered fusion protein comprised of modified IL-2 designed to selectively expand anti-tumor T cells and NK cells while avoiding activation of immunosuppressive cells [181].
NCT04477876 Cutaneous melanoma	Anti-CD160-TM agonist antibody	Transmembrane isoform of CD160 (CD160-TM) is expressed on activated NK cells. Binding of agonist antibody to CD160-TM can promote NK cell dependent anti-tumor effect [182]
NCT03420963 Phase I Advanced malignancy, including cutaneous melanoma	Ex-vivo expanded allogeneic NK cells	Cord Blood-derived Expanded Allogeneic NK cells are cord-blood derived, expanded ex-vivo and administered to patients after pre-treatment with etoposide and cyclophosphamide
